# Body image dissatisfaction and emotional eating across age and weight status: a comparative cross-sectional study

**DOI:** 10.3389/fpsyt.2026.1916354

**Published:** 2026-09-21

**Authors:** Anna Guerrini Usubini, Adele Bondesan, Diana Caroli, Francesca Frigerio, Laura Abbruzzese, Ferruccio Nibbio, Paolo Fanari, Gianluca Castelnuovo, Alessandro Sartorio

**Affiliations:** 1 Experimental Laboratory for Auxo-Endocrinological Research, Istituto Auxologico Italiano, Istituto di Ricovero e Cura a Carattere Scientifico (IRCCS), Piancavallo-Verbania, Italy; 2 Division of Auxology, Istituto Auxologico Italiano, Istituto di Ricovero e Cura a Carattere Scientifico (IRCCS), Piancavallo-Verbania, Italy; 3 Division of Cardiological Rehabilitation, Istituto Auxologico Italiano, Istituto di Ricovero e Cura a Carattere Scientifico (IRCCS), Piancavallo-Verbania, Italy; 4 Division of Pneumological Rehabilitation, Istituto Auxologico Italiano, Istituto di Ricovero e Cura a Carattere Scientifico (IRCCS), Piancavallo-Verbania, Italy; 5 Psychology Research Laboratory, Istituto Auxologico Italiano, Istituto di Ricovero e Cura a Carattere Scientifico (IRCCS), Milan, Italy

**Keywords:** aging, body image, body image dissatisfaction, emotional eating, obesity

## Abstract

**Background:**

Obesity in older adults is associated with significant medical, functional, and psychological consequences, including body image dissatisfaction and maladaptive eating behaviors such as emotional eating. Although these associations have been extensively studied in younger populations, limited evidence is available in older adults with obesity. This study aimed to examine differences in body dissatisfaction by age and weight status and its association with emotional eating in adults and older adults with obesity.

**Methods:**

108 participants were included and divided into four subgroups based on age and weight status. Body dissatisfaction was assessed using the Body Uneasiness Test (BUT-A). Emotional eating was evaluated using the Emotional Eating subscale of the Dutch Eating Behaviour Questionnaire (EE-DEBQ). Two-way ANOVAs were conducted to examine the effects of age, weight status, and their interaction on body dissatisfaction, while Pearson correlations were used to assess associations between body dissatisfaction and emotional eating.

**Results:**

The analysis showed significant main effects of both age and weight status on global body dissatisfaction, with higher BUT-GSI scores observed in adults than in older adults and in participants with obesity compared with normal-weight individuals. Across all BUT dimensions, participants with obesity reported higher levels of body image concerns, avoidance, compulsive self-monitoring, and depersonalization. Significant age × weight status interactions emerged for weight phobia and body image concerns. Age significantly affected body image concerns and avoidance. Positive correlations between body dissatisfaction and emotional eating were found in adults with obesity (r=0.443, p=0.024) and older adults with obesity (r=0.480, p=0.004), as well as in normal-weight adults (r=0.573, p=0.001).

**Conclusion:**

The findings highlight the significant role of both ageing and obesity in shaping body image experiences.

## 1 Introduction

Obesity in older adults is an increasingly prevalent public health concern and is associated with substantial medical, functional, and psychosocial burden. Beyond medical complications (1, 2), obesity in later life contributes to disability, reduced mobility, loss of independence, and poorer quality of life (3). At the same time, ageing is characterized by significant body composition and functional changes, including increased fat mass, reduced muscle mass and strength, altered physical appearance, and a progressive decline in physical performance. These age-related modifications may significantly affect body perception and the subjective experience of one’s own body, particularly among individuals living with obesity (3).

Body image is defined as the multidimensional representation that individuals hold of their own bodies, encompassing perceptions, thoughts, emotions, and behaviors related to physical appearance (4). Recent models conceptualize body image as a complex construct involving cognitive, emotional, and behavioral dimensions, with important implications for psychological well-being and eating-related behaviors (5, 6). Within this framework, body image disturbances refer to persistent negative evaluations of one’s body, including body dissatisfaction, overvaluation of weight and shape, body shame, and distorted body-related self-perceptions.

Extensive literature has demonstrated that body dissatisfaction is strongly associated with lower self-esteem, depressive and anxiety symptoms, emotional dysregulation, and impaired psychosocial functioning (7). Moreover, body dissatisfaction has been consistently identified as a transdiagnostic risk factor for the development and maintenance of disordered eating behaviors (8, 9). Individuals with overweight and obesity tend to report particularly high levels of body dissatisfaction, often driven by weight stigma, internalization of sociocultural appearance ideals, and repeated experiences of discrimination and negative social evaluation (10). These experiences may reinforce feelings of shame and inadequacy, thereby increasing psychological distress and vulnerability to maladaptive coping strategies (11).

One of the most relevant maladaptive responses associated with body dissatisfaction is emotional eating, defined as the tendency to consume food in response to negative emotional states—such as stress, anxiety, sadness, and loneliness—rather than physiological hunger cues (12). Emotional eating has been consistently linked to difficulties in emotion regulation, depressive and anxious symptomatology, and increased risk of weight gain and obesity (12–14). Previous studies suggest that individuals experiencing greater body dissatisfaction are more likely to engage in emotional eating and binge-eating behaviors as dysfunctional strategies to cope with negative affect and body-related distress (15). Despite growing evidence supporting these associations, most research on body image and emotional eating has historically focused on adolescents and younger adults, predominantly women. However, a higher prevalence of body dissatisfaction was found among older women with overweight and obesity (16–18).

Given these considerations, the present study aimed to investigate body image dissatisfaction and its association with emotional eating in older adults with obesity.

## 2 Materials and methods

### 2.1 Participants and procedures

A total of 108 participants were included in the study and divided into four independent subgroups according to age and weight status: normal-weight adults (n=29; mean age ± SD: 42.8 ± 6.1 years; mean body mass index: BMI ± SD: 22.1 ± 2.2 kg/m²); adults with obesity (n=26; mean age ± SD: 44.0 ± 4.8 years; mean body mass index: BMI ± SD: 48.8 ± 7.0 kg/m²); normal-weight older adults (n=19; mean age ± SD: 69.5 ± 5.9 years; mean body mass index: BMI ± SD: 22.8 ± 2.3 kg/m²); older adults with obesity (n=34; mean age ± SD: 70.0 ± 4.5 years; mean body mass index: BMI ± SD: 42.9 ± 10.8 kg/m²).

Participants with obesity (adults and older adults) were consecutively recruited at Istituto Auxologico Italiano, IRCCS, Piancavallo-Verbania, a third-level medical center specialized in the rehabilitation of severe obesity and eating disorders. The inclusion criteria were: (1) being Italian; (2) aged between 18 and 64 years for adults and 65 and 80 years for older adults; (3) having a BMI ≤ 24 for normal-weight participants and a BMI ≥ 30 for participants with obesity. For all groups, the exclusion criteria included any psychiatric disorders and/or physical or mental impairments that could compromise the understanding of the questionnaires (such as visual blindness).

After being informed about the research and providing written informed consent to participate, the participants were asked to provide their socio-demographic data and complete self-report questionnaires. The study was approved by the Territorial Ethical Committee (CET 5), Lombardy Region, Italy (approval date: April 1st, 2025; approval number: 130/25; research code: 01C509; acronym: OBEQOL). The research was conducted in accordance with the Declaration of Helsinki and its subsequent revisions.

### 2.2 Measures

For participants with obesity (adults and older adults), internal medical staff measured weight and height to calculate Body Mass Index using the appropriate formula (kg/m2). Standing height was determined using a Harpenden Stadiometer (Holtain Limited, Crymych, Dyfed, UK). Weight was measured to the nearest 0.1 kg using an electronic scale (RoWU 150, Wunder Sa.bi., Trezzo sull’Adda, Italy). For normal-weight participants (adults and older adults), weight and height were measured by internal medical staff (when possible for subjects recruited among medical, research, and administrative staff) and/or their general practitioners (for subjects recruited among friends and acquaintances). All participants provided demographic information (such as gender and age) by completing a self-report form. Other clinical data were obtained using the following questionnaires:

Body Uneasiness Test (BUT; 20). The questionnaire is widely used in both clinical and non-clinical populations to assess body uneasiness and symptoms associated with eating disorders. It consists of 71 items divided into two sections: BUT-A, which assesses general body uneasiness across five dimensions: weight phobia (WP), body image concerns (BIC), avoidance (A), compulsive self-monitoring (CSM), and depersonalization (D), and a general Global Severity Index (GSI), and BUT-B, which evaluates specific concerns related to particular body parts and bodily functions. Items are rated on a Likert scale, allowing the calculation of both global and subscale scores. Only BUT-A was considered for the current study. Higher scores on all scales indicate greater levels of body uneasiness. The Italian version of the BUT (19) demonstrated good psychometric properties, including high internal consistency and adequate test–retest reliability. Exploratory and confirmatory factor analyses supported the instrument’s multidimensional structure, while correlations with measures of eating psychopathology and body image disturbance confirmed its convergent and discriminant validity. In our sample, the Cronbach’s Alpha of the GSI score was 0.969.

Emotional Eating subscale of the Dutch Eating Behaviour Questionnaire (EE-DEBQ; 21). The questionnaire was originally developed by van Strien and colleagues to evaluate the tendency to eat in response to emotional states rather than physiological hunger. The subscale consists of 13 items assessing eating behaviors associated with a range of negative emotions, including anxiety, sadness, anger, loneliness, and boredom. Participants rate each item on a 5-point Likert scale ranging from 1 (“never”) to 5 (“very often”). Higher scores indicate greater levels of emotional eating. The Italian version of the DEBQ (20) has demonstrated satisfactory psychometric properties in both clinical and non-clinical populations. In our sample, Cronbach’s Alpha of the Emotional Eating subscale of the DEBQ was 0.944.

### 2.3 Statistical analysis

The sample size was determined through an *a priori* power analysis using G*Power software (3.1.9.7). Analysis of variance was selected to compare 4 independent groups, with a significance level (α) of 0.05, a statistical power (1−β) of 80%, and a medium effect size. The calculation indicated a required total sample size of 108 participants (27 participants per group) to detect statistically significant differences among the groups.

Descriptive statistics, including means, standard deviations, frequencies, and percentages (depending on the nature of the variables), were computed to summarize the sample characteristics. Differences between males and females in the BUT Global Severity Index (GSI) were explored with an independent-samples *t*-test. We conducted statistical analyses using a General Linear Model (GLM) two-way analysis of variance (ANOVA) in Jamovi (version 2.6). The primary analysis examined the effects of age (adults vs older adults) and weight status (normal weight vs obesity) on the BUT Global Severity Index (GSI), as well as their interaction. Before hypothesis testing, the normality assumption was evaluated using skewness and kurtosis, and by inspecting Q–Q plots within each subgroup. Although deviations from normality were observed in several subgroups, particularly among normal-weight participants, the GLM approach was retained due to its robustness to moderate violations of normality. Homogeneity of variances was assessed using Levene’s test. The primary outcome model included main effects of age and weight status, as well as their interaction term (age × weight status), on GSI of BUT. Estimated marginal means were computed for all combinations of factors, and pairwise comparisons were adjusted using Bonferroni correction. Effect sizes are reported as partial eta squared (η²p). Secondary outcome analyses were conducted on BUT subscales (WP, BIC, A, CSM, and D) using the same factorial ANOVA framework. These analyses were considered exploratory. To control for multiple comparisons across subscales, adjusted significance levels were applied using Bonferroni correction. Associations between the GSI of BUT and emotional eating (Emotional Eating subscale of DEBQ) were assessed using Pearson’s r coefficients in all the subgroups of the study. In all analyses, statistical significance was set at p < 0.05.

## 3 Results

### 3.1 Sample characteristics

The sample was composed of 108 participants divided into four independent subgroups according to age and weight status: normal-weight adults (n=29; mean age ± SD: 42.8 ± 6.1 years; mean body mass index: BMI ± SD: 22.1 ± 2.2 kg/m²); adults with obesity (n=26; mean age ± SD: 44.0 ± 4.8 years; mean body mass index: BMI ± SD: 48.8 ± 7.0 kg/m²); normal-weight older adults (n=19; mean age ± SD: 69.5 ± 5.9 years; mean body mass index: BMI ± SD: 22.8 ± 2.3 kg/m²); older adults with obesity (n=34; mean age ± SD: 70 ± 4.5 years; mean body mass index: BMI ± SD: 42.9 ± 10.8 kg/m²).

**Table 1** reports all demographic data.

**Table 1:** Demographic data of the sample.

Variables		Normal-weight adults (n=29)		Adults with obesity (n=26)		Normal-weight older adults (n=19)		Older adults with obesity (n=34)	
		n (%)	M(SD)	n (%)	M(SD)	n (%)	M(SD)	n (%)	M(SD)
Gender	Males	11 (37.93%)		12 (46.15%)		6 (31.57%)		11 (32.35%)	
	Females	18 (62.06%)		14 (53.84%)		13 (68.42%)		23 (67.64%)	
Age (yrs)			42.8 (6.08)	–	44 (4.83)		69.4 (5.87)		70.9 (4.51)
BMI (kg/m^2^)			22.1 (2.24)		48.8 (6.97)		22.8 (2.28)		42.9 (10.78)
Origin	Northern Italy	29 (100%)		25 (96.15%)		19 (100%)		31 (91.17%)	
	Central Italy					–		2 (5.88%)	
	Southern Italy			1 (3.84%)		–		1 (2.94%)	
Educational level	Elementary school	29 (100%)		–		2 (10.52%)		8 (23.52%)	
	Middle school	–		4 (15.38%)		6 (31.57%)		5 (14.70%)	
	High School	–		19 (73.07%)		5 (26.31%)		15 (44.11%)	
	Bachelor	–		3 (11.53%)		6 (31.57%)		4 (11.76%)	
	Master	–		–				2 (5.88)	
Marital status	Single	1 (3.44%)		12 (46.15%)		–		3 (8.82)	
	Cohabitant	17 (58.62)		5 (19.23%)		–		2 (5.88%)	
	Married	7 (24.13%)		7 (26.92%)		16 (84.21%)		17 (50%)	
	Divorced	2 (6.89%)		2 (7.69%)		2 (10.52%)		4 (11.76%)	
	Widower			8 (30.76%)		1 (5.26%)		–	
	Other	2 (6.89%)		–		–		–	
Employment	Worker			5 (19.23%)		1 (5.26%)		–	
	Employed	3 (10.34%)		7 (26.92%)		1 (5.26%)		1 (2.94%)	
	Freelancer	12 (41.37%)		1 (3.84%)		1 (5.26%)		2 (5.88%)	
	Manager	12 (41.37%)		1 (3.84%)		1 (5.26%)		–	
	Retired	2 (6.89%)		1 (3.84%)		8 (42.10%)		31 (91.18)	
	Unemployed	–		11 (42.30%)		7 (36.84%)		–	

BMI, Body Mass Index.

Percentages are within groups.

Gender differences in GSI-BUT scores were examined separately within each age and weight-status subgroup. Among normal-weight adults, women had higher GSI scores than men, although this difference did not reach statistical significance (M = 0.654, SD = 0.854 vs. M = 0.201, SD = 0.146, respectively; *t* = −1.73, *p* = 0.094). A similar pattern was observed among adults with obesity, with women reporting higher GSI scores than men; however, the difference was not statistically significant (*t* = −1.88, *p* = 0.072). Among normal-weight older adults, women had significantly higher GSI scores than men (M = 0.62, SD = 0.72 vs. M = 0.02, SD = 0.03; *t* = −2.97, *p* = 0.012). Conversely, no significant gender difference was observed among older adults with obesity (*t* = −1.33, *p* = 0.192).

**Table 2** reports the distribution of genders and the differences between males and females in GSI across the four subgroups.

**Table 2:** Distribution of genders and the differences between males and females in GSI across the four subgroups.

Subgroups	n (%)	Variables	GSI M(SD)	Independent-samples t-test (*p*)
Normal-weight adults (n=29)	Males Females	11 (37.93) 18 (62.07)	0.201 (0.146) 0.654 (0.854)	-1.73 (0.094)
Adults with obesity (n=26)	Males Females	12 (46.15) 14 (53.85)	1.54 (1.24) 2.36 (0.972)	-1.88 (0.072)
Normal-weight older adults (n=19)	Males Females	6 (31.58) 13 (68.42)	0.02 (0.03) 0.62 (0.72)	-2.97 (0.012)*
Older adults with obesity (n=34)	Males Females	11 (32.35) 23 (67.65)	0.079 (0.77) 1.36 (1.28)	-1.33 (0.192)

*:p<0.05.

Information about the normal distribution of the variables are presented in **Table 3**.

**Table 3:** Normal distribution of the study variables.

Variables	Skeweness	Kurtosis
Age (yrs)	-0.005	-1.490
BMI (kg/m^2^)	0.210	-0.852
Weight Phobia	1.217	0.388
Body Image Concerns	0.980	-0.190
Avoidance	1.445	1.029
Compulsive self-monitoring	1.544	2.414
Depersonalization	1.763	2.311
Global Severity Index	1.208	0.387
Emotional eating	0.626	-0.126

### 3.2 Primary outcome: effects of age (adults vs older adults), weight status (normal weight participants vs participants with obesity), and their interaction (age x weight) on the Global Severity Index of BUT (GSI-BUT).

A two-way GLM ANOVA was conducted to examine the effects of age (adults vs. older adults) and weight status simultaneously (normal weight vs. obesity) on BUT-A GSI scores. The overall model was significant, F(3, 104) = 13.80, p < 0.001, explaining 28.5% of the variance in GSI (R² = 0.285, adjusted R² = 0.264). Weight status had a significant main effect, F(1, 104) = 33.84, p < 0.001, η²p = 0.246, with participants with obesity showing higher GSI scores than normal-weight participants. A smaller but significant main effect of age was also observed, F(1, 104) = 4.87, p = 0.030, η²p = 0.045, indicating higher GSI scores among adults than older adults. The age × weight status interaction approached, but did not reach, conventional statistical significance, F(1, 104) = 3.89, p = 0.051, η²p = 0.036. Inspection of the estimated marginal means suggested that age-related differences in GSI were minimal among normal-weight participants (adults: M = 0.482; older adults: M = 0.437), whereas a larger difference was observed among participants with obesity, with adults having higher GSI scores than older adults. Similarly, the difference between participants with obesity and those with normal weight appeared more pronounced among adults than older adults. This pattern may suggest that the association between obesity status and body image disturbance is stronger among adults; however, this interpretation should be considered cautiously, as the interaction did not reach conventional statistical significance.

### 3.3 Secondary outcomes: effects of age (adults vs older adults), weight status (normal weight participants vs participants with obesity), and their interaction (age x weight) on the BUT subscales.

Several two-way GLM ANOVAs were conducted to examine the effects of age (adults vs older adults) and weight status (normal weight participants vs participants with obesity) on the subscales of BUT-A.

The model on Weight Phobia (WP) was significant, F(3, 104) = 12.22, p < 0.001, explaining 26.1% of the variance (R² = 0.261, adjusted R² = 0.239). The analysis revealed a significant main effect of weight status, F(1, 104) = 28.65, p < 0.001, η²p = 0.216, indicating higher weight phobia among individuals with obesity compared to normal-weight participants. The main effect of age was not significant, F(1, 104) = 2.13, p = 0.147, η²p = 0.020, suggesting no overall differences between adults and older adults in weight-related fear. Importantly, age and weight status showed a significant interaction, F(1, 104) = 5.67, p = 0.019, η²p = 0.052. Inspection of estimated marginal means indicated that among normal-weight participants, WP scores were relatively low and comparable between adults and older adults. In contrast, among participants with obesity, WP was substantially higher in adults compared to older adults, suggesting that weight-related fear is particularly pronounced in adult individuals with obesity, whereas this effect appears attenuated in older adults.

Regarding Body Image Concerns (BIC), the results showed that the overall model was significant, F(3, 104) = 18.44, p <.001, explaining 34.7% of the variance (R² = 0.347, adjusted R² = 0.328). The analysis revealed significant main effects of both age, F(1, 104) = 8.30, p = 0.005, η²p = 0.074, and weight status, F(1, 104) = 41.70, p < 0.001, η²p = 0.286. These results indicate higher body image concerns in adults than in older adults, and substantially higher scores in individuals with obesity compared to normal-weight participants, with a large effect size for weight status. Importantly, a significant interaction between age and weight status was observed, F(1, 104) = 6.87, p = 0.010, η²p = 0.062. Inspection of estimated marginal means indicated minimal age differences among normal-weight participants, whereas a pronounced age-related difference emerged among individuals with obesity, with higher BIC scores in adults compared to older adults. This pattern suggests that concerns related to physical appearance are particularly elevated among adults with obesity, while attenuated in older adults.

The overall statistical model on Avoidance (A) was significant, F(3, 104) = 11.52, p < 0.001, explaining 24.9% of the variance (R² = 0.249, adjusted R² = 0.228). The analysis revealed significant main effects of both age, F(1, 104) = 5.87, p = 0.017, η²p = 0.053, and weight status, F(1, 104) = 28.16, p < 0.001, η²p = 0.213. These findings indicate higher avoidance behaviors in adults compared to older adults, and markedly higher avoidance in individuals with obesity compared to normal-weight participants. The interaction between age and weight status was not statistically significant, F(1, 104) = 2.31, p = 0.131, η²p = 0.022. Examination of estimated marginal means indicated that avoidance levels were relatively low among normal-weight participants regardless of age, whereas higher levels were observed among participants with obesity, particularly among adults compared to older adults. However, this pattern did not reach statistical significance at the interaction level.

For the Compulsive Self-Monitoring (CSM) subscale, the results showed that the overall model was statistically significant, F(3, 104) = 3.53, p = 0.017, explaining a relatively small proportion of variance (R² = 0.093, adjusted R² = 0.066). The analysis showed a non-significant main effect of age, F(1, 104) = 1.20, p = 0.276, η²p = 0.011, indicating comparable levels of compulsive body monitoring in adults and older adults. In contrast, weight status showed a significant main effect, F(1, 104) = 9.74, p = 0.002, η²p = 0.086, with higher CSM scores among individuals with obesity than among normal-weight participants. The interaction between age and weight status was not significant, F(1, 104) = 0.24, p = 0.628, η²p = 0.002. Inspection of estimated marginal means confirmed that compulsive self-monitoring was relatively low in normal-weight participants regardless of age, whereas higher levels were observed in participants with obesity, without meaningful influence by age.

Finally, results on Depersonalization (D) showed that the overall model was significant, F(3, 104) = 8.53, p <.001, explaining 19.7% of the variance (R² = .197, adjusted R² = .174). The analysis showed a non-significant main effect of age, F(1, 104) = 2.63, p = 0.108, η²p = 0.025, indicating comparable levels of depersonalization between adults and older adults. In contrast, a significant main effect of weight status emerged, F(1, 104) = 23.71, p < 0.001, η²p = 0.186, with higher depersonalization scores among individuals with obesity than among normal-weight participants. The interaction between age and weight status was not significant, F(1, 104) = 0.55, p = 0.462, η²p = 0.005. Estimated marginal means indicated low levels of depersonalization in normal-weight participants, whereas participants with obesity showed higher levels, with a similar pattern across age subgroups.

Means and standard deviations for all the scales among the four subgroups were presented in **Table 4**.

**Table 4:** Means and standard deviations for all the scales among the four subgroups.

Variables	Subgroups	N	Mean	SD	Min-Max
Weight Phobia	Normal-weight adults	29	0.431	0.728	0.00-3.13
	Normal-weight older adults	19	0.645	0.975	0.00-3.13
	Adults with obesity	26	2.226	1.352	0.25-4.63
	Older adults with obesity	34	1.335	1.424	0.00-5.00
Body Image Concerns	Normal-weight adults	29	0.690	0.669	0.11-3.33
	Normal-weight older adults	19	0.632	0.843	0.00-2.44
	Adults with obesity	26	2.714	1.548	0.00-5.00
	Older adults with obesity	34	1.487	1.208	0.00-4.11
Avoidance	Normal-weight adults	29	0.397	0.908	0.00-4.67
	Normal-weight older adults	19	0.202	0.492	0.00-2.00
	Adults with obesity	26	1.872	1.400	0.00-4.17
	Older adults with obesity	34	1.020	1.216	0-00-4.17
Compulsive self-monitoring	Normal-weight adults	29	0.497	0.903	0.00-4.40
	Normal-weight older adults	19	0.389	0.685	0.00-2.20
	Adults with obesity	26	1.131	0.919	0.00-3.60
	Older adults with obesity	34	0.853	0.963	0.00-3.60
Depersonalization	Normal-weight adults	29	0.310	0.620	0.00-3.17
	Normal-weight older adults	19	0.140	0.325	0.00-1.00
	Adults with obesity	26	1.391	1.146	0.00-4.00
	Older adults with obesity	34	0.936	1.281	0.00-4.17
Global Severity Index	Normal-weight adults	29	0.482	0.707	0.03-3.65
	Normal-weight older adults	19	0.437	0.661	0.00-2.12
	Adults with obesity	26	1.984	1.158	0.12-4.09
	Older adults with obesity	34	1.178	1.160	0.00-3.08
Emotional eating	Normal-weight adults	29	1.029	0.851	0.00-3.08
	Normal-weight older adults	19	0.794	0.819	0.00-2.77
				
	Adults with obesity	26	1.701	0.997	0.08-4.00
	Older adults with obesity	34	1.403	1.011	0.00-3.85

SD, standard deviation.

### 3.4 Correlations between body dissatisfaction (GSI-BUT) and emotional eating (EE-DEBQ).

Correlation analyses revealed a significant positive association between body dissatisfaction (BUT-GSI) and emotional eating (EE-DEBQ) in adults and older adults with obesity, as well as in normal-weight adults. Specifically, moderate positive correlations emerged in adults with obesity (r = 0.443, p = 0.024) and in older adults with obesity (r = 0.480, p = 0.004), indicating that greater body-related distress was associated with higher levels of emotional eating among participants with obesity, regardless of age. A stronger association was observed in normal-weight adults (r = 0.573, p = 0.001), suggesting that emotional eating may also represent an important maladaptive coping strategy in individuals without obesity when body dissatisfaction is elevated.

In contrast, the correlation observed in normal-weight older adults was weaker and not statistically significant (r = 0.327, p = 0.172). This finding may suggest that, in older age and in the absence of obesity, emotional eating is less strongly linked to body dissatisfaction. Results are presented in **Table 5**.

**Table 5:** Correlations between the global severity index of BUT and emotional eating in all the subgroups.

Subgroup	Pearson’s *r*	*p*
Normal-weight adults	0.573	0.001*
Adults with obesity	0.443	0.024*
Normal-weight older adults	0.327	0.172
Older adults with obesity	0.480	0.004*

*:p<0.05.

## 4 Discussion

The present study aimed to investigate body image dissatisfaction and its association with emotional eating in older adults with obesity. To do so, comparisons among four subgroups of normal-weight adults, adults with obesity, normal-weight older adults, and older adults with obesity in their levels of body dissatisfaction and exploration of the association between body dissatisfaction and emotional eating were performed.

The primary analysis of the GSI-BUT showed significant main effects for both age and weight status, indicating higher body dissatisfaction in adults than in older adults and markedly higher levels of body dissatisfaction in individuals with obesity than in normal-weight participants. This is consistent with recent literature suggesting that body dissatisfaction remains prevalent across the lifespan but tends to manifest differently according to developmental stage and weight status (21). The age-related differences observed in the present study align with lifespan models of body image, which propose that appearance-related concerns may gradually decline with advancing age as individuals report increasing value on body functionality, health, and psychosocial well-being than on physical attractiveness (22). Nevertheless, it is important to note that body dissatisfaction remains highly prevalent among older populations, particularly among women, indicating that ageing does not completely protect against negative body image experiences (23).

The strong main effect of weight status is consistent with a substantial body of literature identifying obesity as one of the most robust correlates of body dissatisfaction (24). Individuals with obesity are frequently exposed to weight-related stigma (25), internalized negative stereotypes, and societal pressures favouring thinness, all of which contribute to heightened body image concerns and psychological distress (26). Recent reviews have emphasized that body dissatisfaction represents a central psychosocial burden associated with obesity and is closely linked to poorer mental health outcomes, reduced self-esteem, maladaptive eating behaviors, and diminished quality of life (27, 28).

Secondary analyses of the BUT subscales provided a deeper understanding of the relationship between age, weight status, and specific dimensions of body image disturbance. Consistent with findings for the global GSI score, weight status emerged as a significant factor across all BUT subscales, with individuals with obesity reporting higher levels of dissatisfaction, body-related concerns, avoidance behaviors, compulsive self-monitoring, and fear of weight gain than normal-weight participants. These results are consistent with previous research indicating that obesity is associated not only with elevated levels of global body dissatisfaction but also with a wide range of maladaptive cognitive, emotional, and behavioral responses toward the body, including negative body-related cognitions, body shame, emotional distress, body avoidance, and disordered eating behaviors (10).

Age-related differences emerged specifically in the Body Image Concerns and Avoidance subscales, with adults reporting higher levels of appearance-related preoccupation, compulsive body monitoring, and avoidance behaviors than older adults. This finding aligns with lifespan theories of body image, which propose that appearance investment gradually decreases across adulthood, whereas body functionality, health, and psychosocial well-being become increasingly important sources of self-worth and body evaluation (28, 29).

Correlations between the Global Severity Index of BUT and emotional eating in all the subgroups of the sample (adults with obesity, older adults with obesity, normal-weight adults, normal-weight older adults) showed a significant positive association between the GSI of BUT and emotional eating in adults and older adults with obesity, indicating that the higher the level of body dissatisfaction, the higher the level of emotional eating, among participants with obesity (both in adults and older adults).

These findings align with contemporary models of emotional eating, which conceptualize eating behavior as a maladaptive emotion-regulation strategy triggered by negative affective states, including body-related distress (12–19, 30). Within this framework, body-related distress may represent a salient source of negative affect that increases vulnerability to emotional eating. Individuals who experience elevated body dissatisfaction are more likely to use eating as a coping mechanism for managing feelings of shame, frustration, anxiety, and self-criticism arising from perceived discrepancies between their actual and ideal body image (31).

Interestingly, there was also a strong association between GSI and emotional eating in normal-weight adults, which was even stronger than that observed in the two groups with obesity. One possible explanation is that, among individuals with normative weight, body dissatisfaction may be particularly closely linked to subjective self-evaluation and internalization of sociocultural appearance ideals, rather than to objective weight status per se. In this context, body-related distress, including shame, self-criticism, and perceived discrepancies between actual and ideal body image, may represent a salient source of negative affect and increase reliance on eating as a maladaptive emotion-regulation strategy. Thus, the observed association suggests that the psychological meaning attributed to body shape and appearance may be particularly relevant to emotional eating even in the absence of obesity. This finding suggests that emotional eating may not be exclusively related to obesity but may also represent an important coping strategy among individuals who experience elevated body dissatisfaction despite having a normative weight status. Such a pattern is consistent with cognitive-behavioral models of body image, which posit that subjective body image evaluations, rather than objective body weight alone, are central determinants of psychological distress and maladaptive eating behaviors (7). Indeed, a growing body of evidence indicates that individuals with normal weight who strongly internalize sociocultural appearance ideals may experience substantial body dissatisfaction, which in turn increases vulnerability to emotional eating, disordered eating attitudes, and other dysfunctional weight-control behaviors (32). These findings support the notion that perceived discrepancies between one’s actual and ideal body image, rather than weight status per se, may play a critical role in the development of maladaptive eating patterns (12).

In contrast, the correlation between GSI and emotional eating observed in normal-weight older adults was weaker and not statistically significant. This may reflect an age-related shift in body image investment. Lifespan perspectives suggest that appearance-related concerns tend to become less central to self-evaluation with advancing age, while body functionality, health, and psychosocial well-being gain greater importance, thereby reducing the influence of body dissatisfaction on eating behavior (22).

In conclusion, the findings of the present study highlighted the significant role of both age and weight status in shaping body dissatisfaction. Individuals with obesity consistently reported greater body dissatisfaction across all dimensions of the BUT, confirming the strong association between obesity and negative body image. At the same time, adults showed higher levels of global body dissatisfaction, body image concerns, and avoidance behaviors compared to older adults. Importantly, the interaction effects (weight x age) observed for Weight Phobia and Body Image Concerns indicate that obesity affects body image in adulthood, whereas differences between normal-weight individuals and those with obesity appear less pronounced in older age.

From a clinical perspective, the findings of the present study highlight the importance of considering both age and weight status when assessing body image disturbances. While obesity appears to represent a pervasive risk factor across multiple domains of body dissatisfaction, adulthood may constitute a particularly vulnerable period for the development of weight-related fears, appearance preoccupations, and body-focused avoidance behaviors. Interventions targeting body image in individuals with obesity may therefore benefit from adopting a lifespan perspective, with greater emphasis on reducing body surveillance, appearance-based self-evaluation, and weight-related anxiety among adults, while addressing age-specific concerns related to body functioning and well-being in older populations.

The study has several strengths. One of the main strengths of the present study is its focus on an understudied population, namely older adults with obesity, a subgroup that has received limited attention in research on body image disturbances and emotional eating. While previous literature has extensively examined these psychological processes in adolescents and younger adults, little is known about how body dissatisfaction and emotionally driven eating behaviors manifest and interact in later life. By adopting an integrated perspective that combines psychological, behavioral, and ageing-related dimensions, this study contributes to a more comprehensive understanding of the emotional experience of obesity in older age. Furthermore, the study highlights the importance of considering body image concerns not only as issues affecting younger populations but also as clinically relevant factors influencing mental health and eating behaviors among older individuals.

Despite its contributions, the present study has several limitations that warrant acknowledgement. First, the cross-sectional design precludes causal inference from the observed associations between body dissatisfaction and emotional eating. Although we found significant correlations, we cannot determine whether body dissatisfaction contributes to emotional eating, whether emotional eating exacerbates body dissatisfaction, or whether both are influenced by other underlying psychological factors. Second, all variables were assessed through self-report questionnaires. Consequently, response biases, including social desirability, may affect the findings. Future studies could benefit from incorporating multimethod assessments, including clinical interviews or behavioral measures. Third, the sample size within each subgroup may have limited the statistical power to detect smaller effects, particularly regarding interaction effects and subgroup correlations. Fourth, the study focused primarily on age and weight status and did not consider other factors that may influence body dissatisfaction and emotional eating. In this regard, the present study initially examined gender differences in GSI-BUT scores. The results suggested a tendency toward greater body image dissatisfaction among women across subgroups; however, this difference reached statistical significance only among normal-weight older adults. These results should nevertheless be interpreted cautiously because of the relatively small sample sizes within each subgroup and the unequal distribution of men and women, particularly among normal-weight older adults (6 men vs 13 women) and older adults with obesity (11 men vs 23 women). These small, unbalanced subgroup sizes may reduce statistical power and increase uncertainty in the estimated sex differences. Therefore, the absence of statistically significant differences in the other subgroups should not necessarily be interpreted as evidence of equivalent levels of body image dissatisfaction between men and women. In addition, the sample size did not allow the inclusion of additional variables in the GLM, which may have limited the ability to adequately account for other potential factors associated with body image dissatisfaction. Variables such as socioeconomic status, educational level, physical health conditions, depressive symptoms, anxiety, loneliness, and experiences of weight stigma may play an important role and should be examined in future research with an adequate sample size.

Additionally, the sample characteristics may limit the generalizability of the findings. Participants were recruited from a specific context and may not represent the broader population of adults and older adults with obesity. In addition, recruiting the control group from different sources, including hospital staff, friends, and family physicians, may have introduced selection bias due to potential differences in characteristics between individuals recruited through different channels. This issue should be considered when interpreting the observed between-group differences and may limit the findings’ generalizability. Future studies involving larger samples would help to increase external validity.

## Data Availability

The original contributions presented in the study are publicly available. This data can be found here: Zenodo, https://doi.org/10.5281/zenodo.22744467.
